# Outcome of right ventricular microaxial pump support in patients undergoing cardiac surgery

**DOI:** 10.1038/s41598-024-58602-w

**Published:** 2024-04-06

**Authors:** Medina Marta, Mahmoud Zada, Nils Theuerkauf, Georg Daniel Duerr, Sebastian Zimmer, Hendrik Treede, Mehmet Oezkur

**Affiliations:** 1 Department of Cardiovasular Surgery, University Hospital of Mainz, University Medical Center of the Johannes Gutenberg University Mainz, Langenbeckstrasse 1, 55131 Mainz, Germany; 2 Department of Anesthesiology and Intensive Care Medicine, University Hospital of Bonn, Bonn, Germany; 3 Department of Cardiology and Rhythmology, Hospital Mechernich, Mechernich, Germany; 4 Department of Cardiology, University Hospital of Bonn, Bonn, Germany

**Keywords:** Cardiac device therapy, Heart failure

## Abstract

Right ventricular failure (RVF) after cardiac surgery is associated with an in-hospital mortality rate of up to 75%. Microaxial flow pumps are one of the mechanical circulatory supports (MCS) options available for the treatment of RVF, however the specifics of timing and indication for MCS, as well as predictors for survival, remain unclear due to a dearth of published data. We evaluated the clinical outcome of patients treated with Impella-RP for predictors of mortality and the hemodynamic effects of the pump. This is a single-center retrospective observational study involving adult patients who underwent cardiac surgery with cardiopulmonary bypass between January 2019 and December 2020 in cardiac surgery and required therapeutic management of RVF with an Impella-RP. Overall, 18 patients were included and analyzed for factors that could be associated with mortality, or that could be predictors of patient outcomes for this population. Treatment of RVF with Impella-RP improved the patient hemodynamics significantly and had a survival rate of 61% within 30 days. Patients with isolated CABG or better liver function before implantation had a better survival rate, which may indicate that underlying disease and timing of implantation are significant for successful treatment of RVF.

## Introduction

Right ventricular failure (RVF) is a rare condition, but it is associated with high mortality rates^1,2^. Furthermore, RVF incidence may be underreported as the mechanisms underlying RVF are unclear and diagnosis is difficult^3^. Following conventional cardiac surgery, the prevalence of RVF is reported to be between 0.5 and 3%, while the incidence after left ventricular assist device (LVAD) implantation is reported with 15% to 25% of all LVAD cases^4^.

Conservative treatment with inotropic or nitric oxide therapies has no significant effects on survival, although nitric oxide in particular is associated with hemodynamic improvement. In the last decade, mechanical circulatory support (MCS) of the right ventricle (RV) was introduced as a "bridging" intervention for faster recovery, offering an opportunity to rapidly stabilize patients with cardiogenic shock involving the RV.

The Impella-RP (Abiomed, Danvers, MA) is a minimally invasive percutaneous RV assist device for the treatment of RVF, and the only model approved by the Food and Drug Administration. The RECOVER RIGHT study demonstrated the safety and efficacy of Impella-RP in supporting patients with RVF, enabling early recovery^5^. However, data regarding the Impella-RP remains limited due to low implantation rates; to date, there have been only three multicentric publications and one single center experience^2,6–8^.

The survival rate after Impella-RP support varies based on the indication for implantation and severity of shock; as such, it may range from 28.6 to 49%.^2^ It is therefore important to determine which patients may benefit from Impella-RP use and what factors determine a better outcome after Impella-RP support^2^.

The aim of this study was to evaluate the clinical outcome of patients treated with Impella-RP after cardiac surgery, examining the hemodynamic effects and determining predictors of mortality in the largest cohort in Europe.

## Methods

### Study population

In this retrospective observational cohort study, all patients undergoing cardiac surgery with cardiopulmonary bypass and who developed post-operative RVF between January 2019 and December 2020 at University Hospital Bonn were screened for study inclusion. A total of 24 patients were treated with Impella-RP, though 3 patients were excluded due to Impella-RP dislocation with a treatment duration < 6 h. Three more patients had a BiPella treatment (left and right ventricular Impella at the same time due to biventricular failure). Those patients were excluded as well. As such, the study population included 18 adult patients evaluated through medical record review and descriptive analysis. This study was approved by the Rheinische Friedrich-Wilhelms-Universität Bonn ethics committee. (Ethics approval Number: 168/20) An informed consent for the retrospective analysis of the data was obtained from all subjects. All methods were performed in accordance with the relevant guidelines and regulations for good clinical and good scientific practice.

The indication for Right ventricular microaxial flow pump implantation was the standard of care for isolated RVF. The definition of RVF was an elevated lactate levels over 8 mmol/l, vasoconstrictors > 0.1 γ and at least one inotropic drug, with a central venous pressure (CVP) > 15 mmHg. Furthermore, a pulmonary catheter was introduced in each case and other reversible causes for cardiogenic shock were ruled out with transesophageal echocardiography.

The implantation of Impella-RP was performed under fluoroscopy using a pigtail catheter to introduce the placing wire in the pulmonary artery in the operating room or catheterization lab. All patients were anticoagulated with intravenous heparin with an activated clotting time aim of 160–180.

### Study endpoints

The primary end point for the study was the survival rate at 30 days after device explantation. Secondary endpoints were the hemodynamic effects of device implantation after initiation of Impella-RP support, decreased use of inotropes during Impella-RP support, and device complications.

### Statistics

Statistical analysis was performed using SPSS (Statistical Package for the Social Sciences) for macOs® version 29.0.1.0 (SPSS Inc., Chicago, Illinois, USA). All data were anonymized and treated according to data protection regulations^19^. The local research ethics committee approved the study.

Continuous variables were expressed as mean ± standard deviation. Normality was tested using the Kolmogorow-Smirnow-Test. Categorical variables were shown as absolute (n) and relative (%) frequencies. Continuous variables with a normal distribution were tested using the Student’s t-test, while those that were not normal were tested with the Mann–Whitney-Test. The non-parametric or categorical data were tested with Pearson chi-square test. Values were considered statistically significant at a probability factor *p* ≤ 0.05, where a *p*-value less than 0.05 was considered statistically significant. We use paired samples t-tests to compare means before and after Impella-RP treatment and determine the hemodynamic effect on the patient.

### Ethical approval

The authors declare that the procedures were followed according to the regulations established by the Clinical Research and Ethics Committee and to the Helsinki Declaration of the World Medical Association.

## Results

### Demographic analysis

The 18 patient study population comprised 83% (n = 15) males and 17% (n = 3) females with a mean age of 65.83 ± 11.82 years (Table 1). Survival rate at 30 days after Impella-RP explantation was 61.1%, with an overall mortality rate of 38.9%.

**Table 1:** Patient characteristics and association with mortality.

	Total (n = 18)	Survival (n = 11)	Death (n = 7)	*p*-value
Age	65.83 ± 11.82	67.45 ± 14.29	63.29 ± 6.57	0.326
Gender, n (%)				
Male	15 (83)	10(66)	5(34)	0.280
Female	3 (17)	1(33)	2(66)	
BMI (kg/m^2^)	26.72 ± 4.65	28.64 ± 4.63	23.71 ± 2.87	0.415
Impella-RP Support (days)	3.75 ± 2.63	4.38 ± 2.63	2.90 ± 2.60	0.215
Hospital stay (days)	37.21 ± 37.42	57.50 ± 38.15	10.17 ± 8.25	**0.007**
Impella-RP Implantation after Surgery (days)	3.56 ± 5.72	5.13 ± 7.53	3.33 ± 4.50	0.483
Surgery, n (%)				
Isolated CABG	5(28)	5(100)	0	**0.036**
Non-isolated CABG	13(72)	6(46)	7(54)	
LVAD-implantation	2(7.7)	1(50)	1(50)	
Valve + /-CABG	8(61.5)	4 (50)	4(50)	
Aortic surgery + /-CABG	3(23)	1(33.3)	2(66.6)	
Diagnosis, n (%)				
PaH before Impella-RP Support				
mPAP > 25mmHg, n (%)	9(50)	7(77.8)	2(22.2)	0.083
PCWP > 15mmHG, n (%)	6(33)	5(83.3)	1(16.6)	
Major Bleeding, n (%)	9 (50)	4 (44)	5 (56)	0.147
AvWS, n (%)	14 (78)	7(50)	7(50)	0.070

BMI Body Max-Index, CABG coronary artery bypass graft, LVAD left ventricular assist device, ICM ischemic cardiomyopathy, VCM valvular cardiomyopathy, DCM dilated cardiomyopathy, PaH pulmonary artery Hypertension, mPAP mean pulmonary arterial pressure, PCWP pulmonary capillary wedge pressure, AvWS Acquired von Willebrand Syndrome.

^a^Categorical variables are expressed as *n*/total (%), and continuous variables are expressed as mean ± standard desviation.

^b^Values were considered statistically significant at a probability factor *p* ≤ 0.05.

Significant values are in [bold].

**Figure 1:**
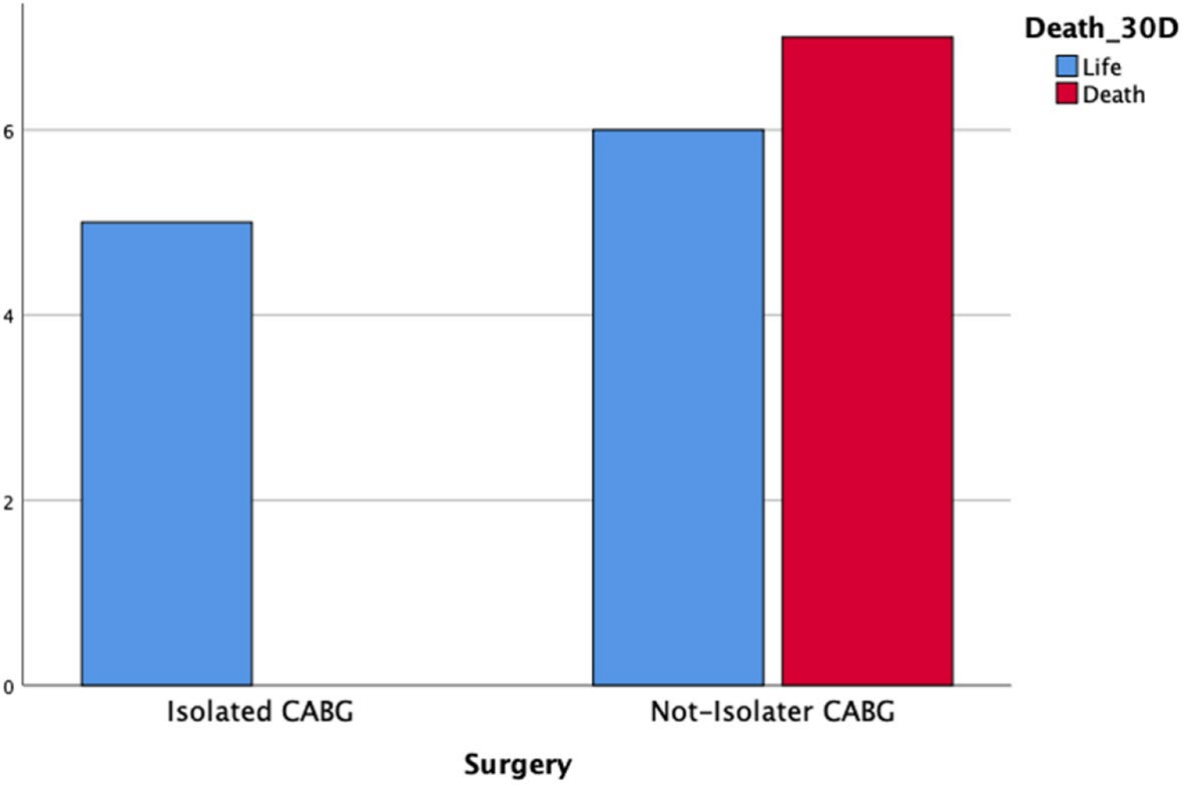
Comparison Survival after postoperative 30-Days. CABG, coronary artery bypass graft.

Demographic data were evaluated in relation to patient mortality, but there were no significant differences. To assess the relevance of the etiology of right heart failure and its impact on the outcome of treatment with Impella-RP, patients were divided according to the surgery received: patients who developed RVF after isolated bypass surgery, and patients who developed RVF after a non-isolated bypass surgery or a surgery other than bypass surgery. Patients undergoing isolated myocardial revascularization surgery had significantly higher survival (odds ratio [OR] = 2.16, 95% confidence interval [95% CI] = 1.2–3.89; *p* = 0.036) after Impella-RP treatment than patients who had undergone valve or combined surgery (Table 1, Fig. 1). Among patients undergoing combined surgeries, 28% (n = 5) required myocardial revascularization, whereas 11% (n = 2) received a left ventricular assist device due to ischemic cardiomyopathy. Importantly, 39% (7 patients) of our cohort exhibited no coronary artery pathology (Table 1).

In general, the timing of Impella RP implantation was very early at our center (3.56 ± 5.72 days) due to adherence to an established Standard Operating Procedure (SOP). Consequently, it is demonstrated that there is no significant statistical relationship with 30-day mortality. Regarding hospital stay, it was significantly longer in those patients who survived right heart failure with Impella-RP support.

Factors such as severe bleeding or the presence of acquired von Willebrand syndrome (AvWs) didn’t demonstrate a significant association with increased risk of death.

### Hemodynamic and liver function analysis

Liver function and lactate dehydrogenase (LDH) levels were evaluated to determine whether there was a significant, and therefore predictive, association between elevated values and patient outcomes (Table 2). However, no statistically significant relationship to mortality rate was detected. Total bilirubin on the last day of Impella-RP treatment appeared to show a trending parameter in relation to mortality rate (*p* < 0.063). When comparing the dynamics of liver function parameters from day zero of Impella-RP implantation to the last day, significant differences were found only in the alanine aminotransferase (ALT) value (*p* < 0.012). This may indicate that patients receiving Impella-RP treatment may be in an advanced stage of RVF at the time of implantation.

**Table 2:** Liver function and hemodynamic parameters during Impella-RP therapy and association with mortality.

Parameter	Total (n = 18)	Survival (n = 11)	Death (n = 7)	*p*-value
Liver				
Total bilirubin, mg/dL				
Day 1	2.43 ± 2.82	1.86 ± 1.48	3.32 ± 4.17	0.299
Day 3	4.6 ± 4.74	3.54 ± 4.47	6.27 ± 5.02	0.245
Last day	5.55 ± 6.02	3.43 ± 4.74	8.87 ± 6.66	0.060
gGT, (U/L)				
Day 1	44.17 ± 27.09	50.64 ± 27.81	34 ± 24.34	0.214
Day 3	80.39 ± 127.79	107.36 ± 159.22	38 ± 24.34	0.274
Last day	66.89 ± 60.4	72.73 ± 56.41	57.71 ± 69.81	0.622
ALT, (U/L)				
Day 1	341.5 ± 1078.64	536.36 ± 1367.61	35.29 ± 15.16	0.352
Day 3	341.94 ± 591.84	467.64 ± 709.34	144.43 ± 281.71	0.271
Last day	254.89 ± 329.83	249.09 ± 317.37	264 ± 374.45	0.929
AST, (U/L)				
Day 1	577.06 ± 1463.6	872.36 ± 1841.2	113 ± 90.82	0.297
Day 3	589.28 ± 759.83	599.18 ± 671.1	573.71 ± 940.6	0.947
Last day	358.22 ± 519.54	328 ± 516.92	405.71 ± 561.36	0.767
LDH, (U/L)				
Day 1	1870.49 ± 1935.35	2489.98 ± 2289.36	941.25 ± 763.32	0.235
Last Day	642.21 ± 487.22	755.85 ± 579.52	471.75 ± 296.41	0.398
Hemodynamic				
PCWP				
Pre-Impella-RP	19 ± 4	17.60 ± 2.30	26	**0.029**
Post Impella-RP	21.43 ± 13.99	25.75 ± 15.58	15.67 ± 11.67	0.394
mPAP				
Pre-Impella-RP	29.27 ± 8.9	29.12 ± 9.41	29.66 ± 9.23	0.934
Post Impella-RP	24.88 ± 9.02	23.43 ± 8.67	35	0.259
Cardiac index				
Pre-Impella RP	2.50 ± 0.80	2.65 ± 0.85	1.96 ± 0.22	0.309
Post Impella RP	3.15 ± 0.75	3.22 ± 0.68	2.9 ± 1.27	0.626

gGT gamma-glutamyl transferase, ALT alanine aminotransferase, AST aspartate aminotransferase, LDH lactate dehydrogenase, PCWP Pulmonary Capillary Wedge Pressure, mPAP Mean pulmonary artery pressure.

^a^Continuous variables are expressed as mean ± standard desviation.

^b^Values were considered statistically significant at a probability factor *p* ≤ 0.05.

Significant values are in [bold].

Furthermore, we did not observe a statistically significant relationship between hemolysis parameters such as LDH and haptoglobin and major bleeding in our patients. Nevertheless, it’s crucial to acknowledge that the retrospective nature of our study prevented the evaluation of other hemolysis parameters that might influence our findings (Table 3).

**Table 3:** Hemolysis parameters and their relation to major Bleeding.

Laboratory on Impella-RP support	Total	No Bleeding	Major Bleeding	p-value
Haptoglobin < 0.1g/dL, n (%)	2 (11.1)	1 (50)	1 (50)	0.765
Bilirrubin total elevated > 1.1 mg/dL, n (%)	17 (94.4)	8 (47.1)	9 (52.9)	0.500

^a^Categorical variables are expressed as *n*/total (%),

^b^Values were considered statistically significant at a probability factor *p* ≤ 0.05.

Though no significant statistical relationship was evinced between hemodynamic values and mortality, a rapid improvement in hemodynamic parameters was observed during treatment with a Impella-RP. Placement of Impella-RP resulted in a significative improvement in the cardiac index (CI) for almost all the patients (mean = − 0.75, 95% CI = − 1.22–0.29; *p* = 0.032; Table 4).

**Table 4:** Paired T-Test for pre- and post Impella-RP hemodynamic parameters.

	Mean	95% CI		*p*-Value
		Lower	Upper	
PCWP	− 6.00	− 18.95	6.95	0.799
mPAP	18.28	0.43	36.14	0.083
CI	− 0.75	− 1.22	− 0.29	**0.032**

PCWP Pulmonary Capillary Wedge Pressure, mPAP mean Pulmonary Artery Pressure, CI cardiac index.

Significant values are in [bold].

**Figure 2:**
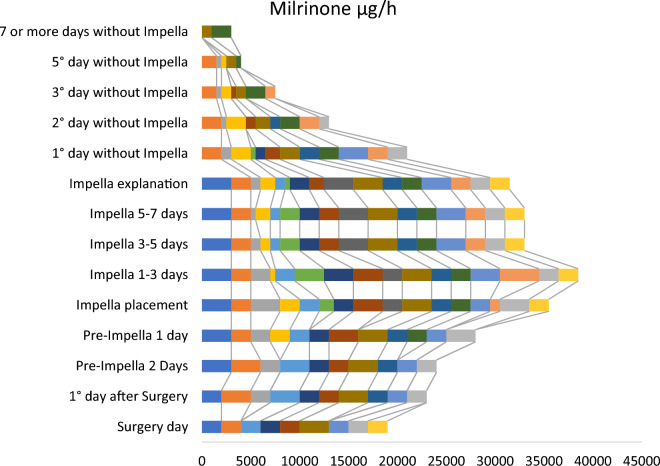
Milrinone interaction during therapy with Impella-RP. Each color denotes an individual patient. Width of each color represents the dosage of Milrinore.

*A*fter Impella-RP implantation, catecholamine support was significantly reduced (Fig. 2). Patients demonstrated a catecholamine peak at the time of Impella placement and at the time of explantation as bridging support, followed by a reduction or discontinuation of catecholamine therapy.

## Discussion

This study is the largest single center retrospective cohort in Europe with Impella-RP for patients with RVF after cardiac surgery. We evaluated which factors provided an approximation of right ventricular function in the postoperative period. Due to the lack of a consensus definition of RVF, and the fact that RVF mechanisms have not been well defined^9^, we evaluated liver function and assessed catecholamine variability as a noninvasive hemodynamic prediction metric. As these factors could be influenced by external complications and were, by nature, secondary assessors for determining right heart function, we performed invasive hemodynamic prediction measurements of CI and mean pulmonary artery pressure (mPAP). We found that patients undergoing isolated myocardial revascularization surgery had significantly higher survival after Impella-RP treatment than patients who had undergone valve or combined surgery, and that Impella-RP therapy significantly improved CI in most patients. We did not find any significant association with mortality for any of the metrics evaluated, though total bilirubin on the last day of Impella-RP treatment and ALT on the first and third days of Impella-RP treatment trended towards significance.

A significant correlation with mortality after 30 days was observed among patients with elevated pulmonary capillary wedge pressure (PCWP) prior to Impella-RP support. It is relevant to note that elevated PCWP levels may indicate severe left ventricular failure or severe mitral stenosis. Among our patients, it is noteworthy that those included in the study had preserved left ventricular function. However, the observation of elevated PCWP levels, which serves as a reasonable surrogate marker of left atrial pressure and left ventricular end-diastolic pressure, suggests that despite compensatory cardiac function, elevated pressures were detected. This indicates a preceding hemodynamic disturbance that could influence the evaluation of these patients, potentially masking global ventricular dysfunction^10,11^.

The use of Impella-RP was most beneficial for patients undergoing isolated myocardial revascularization surgery who develop RVF after open-heart surgery. This might indicate that bridging of ischemia caused RVF to recovery is more likely than non-ischemic RVF. Importantly, despite hemodynamic instability at the time of Impella-RP implantation, these patients developed complete right heart recovery and hemodynamic stability with no mortalities, which was maintained after explantation of the Impella-RP. Impella-RP treatment also resulted in an increased cardiac index in almost all patients, though likely due to the breadth of pathology of these patients, it was not significantly related to survival; regardless, it improved systemic perfusion, enhanced end-organ perfusion, and enabled prompt withdrawal of inotropic and vasopressor support. RVF as a complication after cardiac surgery is challenging to treat and is associated with increased short-term mortality^1,12,13^ and extended hospitalizations; this unsurprisingly results in commensurate cost increases. Thus, implementation of Impella-RP is not only beneficial to improving RV recovery and cardiac output, but it also reduces costs associated with complications from open heart surgery and extended hospitalization times.

The overall mortality evinced by this study is notably lower than that seen in the literature, where overall mortality has reportedly been as high as 51%^2^. In this analysis, overall mortality was closer to that of the RECOVER RIGHT and subsequent conformity assessment and post approval studies (26.7%)^1,5^. It may be that patient selection was important for this, as the cohort in this study contained exclusively patients who underwent cardiac surgery with cardiopulmonary bypass and required therapeutic management of RVF with an Impella-RP. In studies that include consecutive Impella-RP placements, patients could be experiencing RV failure due to numerous conditions, including post-LVAD implantation, acute pulmonary embolism, post-cardiotomy RV failure, post-myocardial infarction shock, and nonischemic cardiomyopathy^2^; it may be that the varied etiologies for RVF result in different therapeutic approaches in addition to MCS. Furthermore, the timing of Impella-RP usage may be crucial to improving mortality outcomes for this population, as this is the case for left ventricular failure MCS^14,15^, and recent work has suggested this holds true for RVF as well^16,17^. Future analyses will need to focus on these factors to ensure the patients who are best served by Impella-RP usage are treated when appropriate for the best odds of survival and recovery.

The principal hemodynamic benefit of Impella-RP is a direct increase in pulmonary artery flow and a concomitant unloading of the RV^6,18^. As described in the RECOVER RIGHT study in 2015^5^, the use of Impella-RP is safe and effective in improving hemodynamics in patients with severe RVF refractory to medical therapy. Given that Impella-RP is a percutaneous right ventricular assist device support, it not only facilitates fast implantation, but also effectively reduces the right ventricular workload without the need for cardiotomy, achieving prompt hemodynamic stabilization of the patient before shock becomes irreversible. Complications such as bleeding or AvWS were managed by clinicians and did not demonstrate increased mortality. Due to the wide range of pathologies of our patients, it is difficult to clarify the specific pathophysiology that results in acute RVF. Factors such as elevated liver function parameters may indicate an advanced stage of RVF at the time of implantation and as such may not be sufficient for treatment with Impella-RP.

Overall, Impella-RP is safe and effective and can be used as standard treatment for a select group of patients. Although our data shows an improvement of hemodynamics in almost all patients, the survival of the patients depends on many other factors. The influence of other factors to mortality has to be addressed in a future prospective study.

## Limitations

The study population was conducted at a single-center with a limited sample size. Due to these factors and the retrospective nature of the study, the results cannot be generalized and it will be necessary to perform further thorough prospective, multicenter research and multivariate analyses multivariate analyses to identify predictors of mortality associated with Impella-RP usage in RVF.

One limitation of our work is that we do not show some baseline patient characteristic, e.g., Cr, LFTs as well as coronary artery or valvular disease, diabetes mellitus or smoking, etc. Also, data on ejection fraction, left and right ventricular dimensions and other morphological or functional parameters, e.g., TAPSE or tricuspid valve regurgitation are not provided.”

## Data Availability

All relevant data are included in the manuscript and its associated files. Due to data protection regulation, we are not allowed to submit the data with the publication. However, in case of a relevant question, the data is available and can be provided by the corresponding author.

## Abbreviations

ALT: Alanine aminotransferase
AvWs: Acquired von Willebrand syndrome
CI: Cardiac index
CVP: Central venous pressure
LDH: Lactate dehydrogenase
LVAD: Left ventricular assist device
MCS: Mechanical circulatory support
mPAP: Mean pulmonary artery pressure
PCWP: Pulmonary capillary wedge pressure
RV: Right ventricle
RVF: Right ventricular failure
